# Intracellular Energy-Transfer Networks and High-Resolution Respirometry: A Convenient Approach for Studying Their Function

**DOI:** 10.3390/ijms19102933

**Published:** 2018-09-26

**Authors:** Marju Puurand, Kersti Tepp, Aleksandr Klepinin, Lyudmila Klepinina, Igor Shevchuk, Tuuli Kaambre

**Affiliations:** Laboratory of Chemical Biology, National Institute of Chemical Physics and Biophysics, Akadeemia tee 23, 12618 Tallinn, Estonia; kersti.tepp@kbfi.ee (K.T.); a.klepinin1989@gmail.com (A.K.); ljudmila.ounpuu@gmail.com (L.K.); igor@chemnet.ee (I.S.) tuuli.kaambre@kbfi.ee (T.K.)

**Keywords:** mitochondria, energy-transfer networks, creatine kinase, adenylate kinase, hexokinase, oxidative phosphorylation, high-resolution respirometry

## Abstract

Compartmentalization of high-energy phosphate carriers between intracellular micro-compartments is a phenomenon that ensures efficient energy use. To connect these sites, creatine kinase (CK) and adenylate kinase (AK) energy-transfer networks, which are functionally coupled to oxidative phosphorylation (OXPHOS), could serve as important regulators of cellular energy fluxes. Here, we introduce how selective permeabilization of cellular outer membrane and high-resolution respirometry can be used to study functional coupling between CK or AK pathways and OXPHOS in different cells and tissues. Using the protocols presented here the ability of creatine or adenosine monophosphate to stimulate OXPHOS through CK and AK reactions, respectively, is easily observable and quantifiable. Additionally, functional coupling between hexokinase and mitochondria can be investigated by monitoring the effect of glucose on respiration. Taken together, high-resolution respirometry in combination with permeabilization is a convenient approach for investigating energy-transfer networks in small quantities of cells and tissues in health and in pathology.

## 1. Introduction

The alterations in cell bioenergetics have become a hallmark of heart diseases and cancer, two of the leading causes of death worldwide. Thus, better knowledge of cellular bioenergetic processes may provide several options for treatment of these diseases. As a part of bioenergetic studies, real-time analysis of oxidative phosphorylation (OXPHOS) with high-resolution respirometry has been extensively applied to investigate mechanisms of this key element of cellular bioenergetics. However, in addition to the ATP synthesis inside mitochondrion, the second and just as important part of the energy provision is the transport of the energy-carrying phosphoryl group from sites of regeneration to ATPases across the cytosol.

Phosphotransfer circuits composed of creatine kinase (CK), adenylate kinase (AK), and glycolytic/glucogenolytic enzymes along with substrate shuttles, such as glycerol-3-phosphate, are essential parts of the cardiac bioenergetic infrastructure and integral to maintaining energy homeostasis [1,2,3]. These phosphotransfer networks are especially necessary for any cell or tissue with high and intermittent energy fluctuations, such as skeletal and smooth muscle, kidney, brain and neuronal cells, retina photoreceptor cells, spermatozoa, and gastric mucosa [4,5,6,7]. The ^18^O phosphoryl oxygen exchange measurements have demonstrated that under basal conditions in intact noncontracting rat diaphragm muscle cells, almost every newly generated ATP molecule appears to be processed by CK (88%) or the AK phosphotransferases prior to its use [8]. In a normal heart, corresponding parameters are 80–88% for CK, about 15% via AK reaction, and the remaining 5–7% via glycolysis [9,10,11].

It is relatively common in the field of bioenergetics to use isolated mitochondria in respirometric studies. This approach enables the assessment of the metabolism inside the mitochondrion e.g., the usage of different respiratory system complexes or detailed assessment of the overall rates of energy production. However, isolation of mitochondria disrupts their normal morphology and interactions with other cellular structures. Furthermore, there is evidence that isolated mitochondria possess several functional characteristics that differ considerably from those of intact mitochondria in permeabilized myofibers and cells [12]. Clearly, the isolated mitochondria do not provide information regarding their function in the intracellular environment under physiological settings, because functional connections between mitochondria and other cellular structures (e.g., ATPases, cytoskeleton), essential for normal function in vivo, are destroyed by the isolation procedure. Therefore, better understanding of bioenergetic processes can be derived from experimental models where the mitochondrial function is directly assessable, but also as undisrupted as possible. In these terms, selective permeabilization of the cellular outer membrane offers several advantages. First, this model preserves the mitochondrial interactions with cellular components existing in vivo as discussed. Secondly, this model enables the assessment of energy-transfer networks connecting mitochondria with ATPases. Thirdly, it allows reduction of necessary sample sizes as compared to that needed for the isolation of mitochondria [13,14]. Therefore, permeabilization offers direct and controllable access to mitochondrial processes in in vivo samples to expand our knowledge beyond data gained from inherently limited in vitro models. Implementation of the permeabilization technique to study OXPHOS in the framework of a molecular system bioenergetics has helped to explain the complex network of cellular bioenergetics in heart muscle cells. Results from these works have demonstrated intracellular diffusion restrictions for metabolites, metabolic compartmentalization, metabolite channeling and functional coupling between energy transport networks and OXPHOS. Characterization of metabolic fluxes, including feedback loops regarding distant energy use back to mitochondria, and complex structure-function relationships between included complexes, resulted in formulation of concept of intracellular energetic units [2,3,15,16,17]. However, for most tissues the intracellular diffusion restrictions for energy metabolites and accompanying micro-compartmentalization together with energy transport circuits is a relatively unexplored and undervalued area in cellular bioenergetics.

In this article, we introduce the possibilities to use high-resolution respirometry to investigate the organization of mitochondrial-cytosolic networks and phosphotransfer networks by using cell permeabilization technique and oxygraphy.

## 2. Organization of Intracellular Environment, Influencing Bioenergetics

### 2.1. Diffusion Restrictions and Micro-Compartmentalization

Effective communication between energy production in mitochondria and energy consumption across the cytosol is vitally important for all cells. Therefore, the diffusion of ADP toward mitochondria is of regulatory importance. Also, ADP is a limiting factor of the ATPase activity and thus it is critical to maintain a high ATP/ADP ratio near the ATPases [4]. Therefore, efficiency of these processes is dependent on the ability of cellular apparatus to remove ADP from the microenvironment of ATPases. In addition, the produced ADP amount should be a signal for sufficient ATP synthesis in mitochondria. In oxidative slow-twitch muscles, such as heart and skeletal muscle, the cross-talk between mitochondrion and ATPases is especially important to avoid disjunction in energy supply and to regulate mitochondrial work at very different levels of energy need.

Paradoxically, in cells, especially in an oxidative muscle cells, the diffusion of molecules, including energy carriers, is much slower than in water due to the diffusion restrictions by organelles and cytoskeleton of the cell. The arrangement of mitochondria in heart cells and skeletal muscle is highly regular which is important for efficient energy transfer to ATPases; in many other tissues the mitochondria are more dynamic [18,19]. In recent decades, several groups have demonstrated that kinetic parameters of energy metabolism measured in isolated mitochondria versus mitochondria in vivo give strikingly different results [20,21,22]. For example, the affinity of mitochondria to exogenous ADP, expressed as apparent Michaelis-Menten constant (*K*m(ADP)), measured in isolated mitochondria from cardiac tissue is approximately 20 µM; the same parameter for mitochondria in vivo, in permeabilized cardiac fibers or cardiomyocytes is close to 400 µM [23]. The *K*m(ADP) value is dependent on muscle type: in glycolytic muscles the mitochondrial affinity for ADP is high, close to this value for isolated mitochondria, while in slow-twitch oxidative muscles it is similar to the heart tissue (*K*m(ADP) = 300–400 µM) as mentioned above [20,21]. Over the last decade studies have demonstrated that the voltage-dependent anion channel (VDAC) regulates the flux of metabolites through the outer mitochondrial membrane (OMM), and is selective for ATP, ADP, AMP, NADH, and NADPH. In the closed state VDAC is virtually impermeable to ATP and ADP [24,25]. Possible explanation for this is that VDAC permeability in muscles is regulated by some cytoskeletal proteins in the level of OMM [26,27,28]. Therefore, the intracellular movement of adenine nucleotides is not as easy as can be expected given their essential biological role.

By using cell permeabilization technique *K*m(ADP) values can be measured in small samples of different tissues or cells which get quick information about the bioenergetics regulation type in this tissue. In biopsies from human gastric mucosa the *K*m(ADP) is about 100 µM [6]. In postoperative samples from colon tumor the affinity of mitochondria to ADP was at the same range (*K*m(ADP) = 126 ± 17 µM) [29,30], whereas human normal colon tissue displayed significantly lower affinity (*K*m(ADP) = 260 ± 55 µM). These data demonstrate that the remodeling of intracellular diffusion barriers is involved in carcinogenesis.

It has been shown that the diffusion restrictions lead to metabolic micro-compartmentalization i.e., unequal concentration distribution of metabolites, including energy metabolites, in different areas of a cell. Spatial micro-compartments are formed where the concentrations of compounds are significantly higher or lower than of the cell in general [31,32,33]. This in turn could lead to situations where local low concentration could hinder cell function as described in cardiac muscle [33,34]. Also, spatial fluctuations in ATP concentrations caused by restricted diffusion raises the need for more efficient energy transport with the opportunity to precise regulation of energy fluxes. This suggests that free diffusion of energy metabolites is ineffective for muscle work and most of the energy flux should be transported by direct transfer through special pathways.

### 2.2. Energy Transport Systems as a Regulator of Oxidative Phosphorylation

Energy channeling through CK and AK transport circuits is more profoundly studied in muscle cells. It was shown twenty years ago that oxidative muscle cells possess strong diffusion restrictions for the ATP and ADP at the level of OMM [20,35]. Unlike ADP and ATP there is no restriction for movement of phosphocreatine (PCr) and creatine through OMM via VDAC [36].

In complete CK energy-transfer systems isoenzymes of mitochondrial CK (MtCK) are functionally coupled to adenine nucleotide translocase (ANT) in the mitochondrial inner membrane compartment. On the other hand, the cytosolic CK isoforms situated near the ATPases create micro-compartments for privileged exchange of substrates and products, and thus assure effective metabolite channeling. ATP input or removal in these micro-compartments will drive the CK reaction predominantly in a given direction. In a particular cell type, at least one dimeric cytosolic isoform is always co-expressed with a MtCK, generally cytosolic muscle type CK (MCK) with sarcomeric MtCK (sMtCK), or cytosolic brain-type CK (BCK) with ubiquitous MtCK (uMtCK) [4,37].

**Figure 1:**
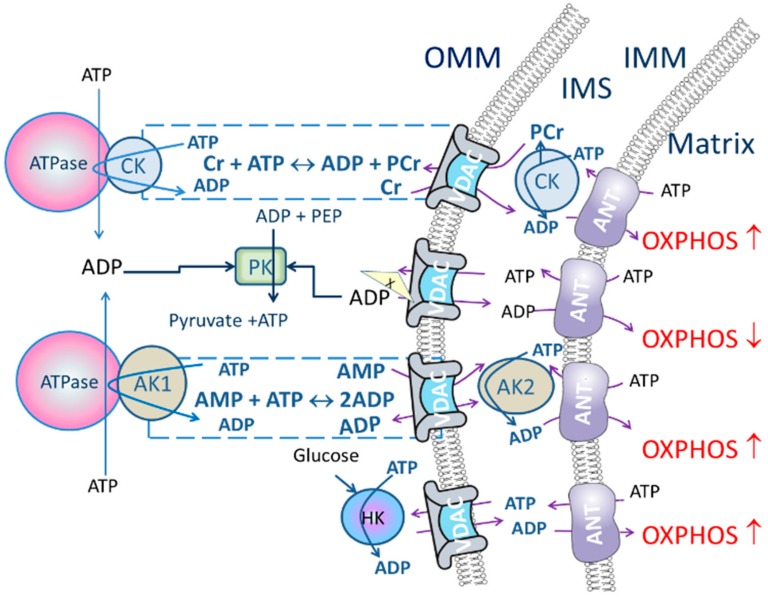
Cellular energy fluxes and transport routes in the context of the current review. In permeabilized cells cytosolic water-soluble liquid components and freely floating proteins are washed out but most of the ATPases and enzymes attached to cytoskeleton or other structures remain active. The movement of the adenine nucleotides through mitochondrial outer membrane (OMM) voltage-dependent anion channel (VDAC) may be restricted by specific protein complex (X). However, the cytosolic creatine kinase (CK) isoforms coupled with ATPase and mitochondrial CK and adenylate kinase (AK) cytosolic isoform AK1 and mitochondrial AK2 create an opportunity for energy transport without ADP and ATP free diffusion in the cytoplasm. In these energy-transfer networks ADP generated in ATPase reactions and ATP produced in mitochondria are quickly directed to CK and AK reactions. Therefore, adding CK and AK activating compounds (creatine (Cr) or AMP, respectively) is also reflected in the rate of oxidative phosphorylation (OXPHOS). In the case of the AK shuttle the AMP derived from AK (AK1) reactions in the cytoplasm enters to the IMS where AK2 converts AMP and ATP to ADP. In addition to speed up the movement of the energy-rich phosphoryl group in cytoplasm, these energy transport systems provide better feedback between ATP consumption and synthesis. Addition of pyruvate kinase (PK) and phosphoenol pyruvate (PEP) to medium traps ADP that is not attached to energy transport systems. Therefore, in the presence of PK-PEP system and without activation of the CK or AK pathway the rate of OXPHOS decreases. Hexokinase (HK) bound to VDAC directs mitochondrial ATP to glycolysis pathway and remained ADP can stimulate OXPHOS. Adenine nucleotide translocase (ANT) is situated in the inner mitochondrial membrane (IMM). IMS, mitochondrial intermembrane space.

In oxidative muscle cells ATP-syntasome [38,39], MtCK and ANT complexes together with respiratory system form a large protein supercomplex called Mitochondrial Interactosome (MI), which regulates not only ATP formation but also its movement out of the mitochondrion [15,36]. The PCr production by MtCK is regulated by ANT, ATP/ADP antiporter, which provides conditions, where PCr formation is kinetically favored. ADP, formed by MtCK, is directly channeled to mitochondrial matrix by ANT where it has an instant influence on the respiration rate (Figure 1). It has also been shown that as the affinity of mitochondrion to ADP is low in oxidative muscle cells due to the restriction of ATP/ADP transport through OMM, the oscillations in the concentration of ADP and creatine in the mitochondrial intermembrane space (IMS) are sufficient to regulate OXPHOS rate [40,41]. Respirometric study of MI, using the method of metabolic control analysis, revealed that the MtCK and ANT complexes are the key points of regulation of respiration rate in cardiac cells under the normal physiological conditions [42].

Regarding to the AK pathway, the isoenzyme AK2 is in IMS [43] and it has been found that ADP generated there from ATP by AK2 can be channeled into mitochondrial matrix by ANT where it stimulates OXPHOS (Figure 1) [44]. This suggests that AK2 plays a role in energy metabolism and energy transfer by regulating the ATP/ADP rate between the cytoplasm, IMS and the mitochondrial matrix. AK2 is strongly expressed in liver, heart, skeletal muscle, and pancreas, but also in kidney, placenta, brain, testis, pancreas, lung, and human gastrointestinal wall [6,29,43,45,46,47]. Moreover, AK2 plays an important role in differentiation of cardiac, neural, and hematopoietic stem cells [47,48,49,50]. It has been shown in Jurkat cell line that induction of apoptosis increased the amount of cytochrome c as well as AK2 in the cytosol [51], therefore evaluation of AK coupling to OXPHOS could be useful for evaluation of cell damage. Also, the possible role for AK2 in the apoptotic process could not be related to the ‘normal function’ of AK in cells but it has been suggested that AK2 is involved in a novel apoptotic pathway by forming a complex with FADD (Fas-Associated protein with Death Domain) and Caspase-10 [52].

The possible cytosolic partner of AK2 in full energy-transfer circuit AK1 is the most abundant AK isoform located in cytosol. It is present in most mammalian tissues, and its expression is especially high in tissues with high energy need, such as brain, skeletal and heart muscles and in erythrocytes [43,46]. The AK1-knockout muscle in mice adapts to the lack of AK1-catalyzed phosphotransfer through up-regulation of glycolytic, CK and guanine nucleotide phosphotransfer systems, but the energetic efficiency of AK1-knockout muscle was lower than that of wild type [53]. The activity of AK in cardiomyocytes develops close to the adult value at the end of the first month already, meanwhile increase in the MtCK isoform content starts in the end of the second postnatal week and the CK pathway is fully developed by the end of third month [54,55]. During aging, decline in the CK pathway is the first detectable sign of the alterations in bioenergetics metabolism in 1-year-old (middle-aged model) rat cardiomyocytes while the alterations in the AK pathway are not significant [55]. Also, Nemutlu et al., using determination of ^18^O labeling ratios in metabolic oligophosphates, detected decrease in CK as well as AK pathway activity in aged rat myocardium. Interestingly, this decrease was found to be smaller in stress conditions (initiated with isoproterenol) [56].

Glycolytic enzymes can also contribute to intracellular high-phosphoryl transfer. Energy-rich phosphoryl groups from ATP can be used to phosphorylate glucose and fructose-6-phosphate near mitochondria and from the other side, in the cytosol pyruvate kinase (PK) can phosphorylate ADP and thereby provide ATP for use (reviewed in [7].

Activity and subcellular localization of HK isoenzymes determines the further metabolic fate (anabolic or catabolic) of glucose-6-phosphate and modulates other intracellular roles of glucose. The tight regulation of HK binding to the OMM depends on cellular energetic needs in skeletal muscle [57]. Also, the decrease in HK2-mitochondrial interaction indicates negative outcome of ischemia-reperfusion injury of the heart [58,59]. Moreover, evidence is mounting that binding of HK2 to VDAC plays a pivotal role in highly malignant cancer cells in promoting cell growth and survival [60,61].

Therefore, studies are needed including not only ATP formation inside mitochondrion, but the interactions of mitochondrion with the other components of the cell to assure transport of phosphoryl group to the energy consumption sites. Besides, energy-transfer pathways work in both directions—to transport energy to the ATPases and to transport information to the mitochondrion. It is important to understand regulatory pathways in bioenergetics and the order of pathological changes to support maintenance of normal functioning of the cells and to start prevention in first signs of alterations.

## 3. Quantitative Assessment of Intracellular Diffusion Restrictions and Energy-Transfer Networks in Permeabilized Cells

### 3.1. Materials and Sample Preparation

Chemicals: All chemicals used in this study were purchased from Roche, Fluka, and Sigma-Aldrich (Saint Louis, MO, USA) only ultra-pure chemicals suitable for molecular biology and work with cell cultures were used.

Mitomed solution: EGTA (0.5 mM), MgCl_2_ (3 mM), K-lactobionate (60 mM), taurine (20 mM), KH_2_PO_4_ (3 mM), sucrose (110 mM), DTT (0.5 mM), HEPES (20 mM), pH 7.1 was used in all respirometry experiments. Supplementation respiratory medium with 5 mg/mL essential fatty acid-free bovine serum albumin (BSA) is recommended.

To make the Mitomed solution dissolve EGTA, MgCl_2_, taurine, KH_2_PO_4_, HEPES, sucrose and add K-lactobionate stock solution (0.5 M, store in 12 mL aliquots at −20 °C); adjust pH to 7.1 with KOH; and store in 25–50 mL aliquots at −20 °C. On day of the experiment weight and add respiratory substrates glutamate/pyruvate and malate; adjust pH to 7.1 with KOH or add 1 M neutralized stock solutions directly into the oxygraph chamber. With added substrates the Mitomed solution can be stored two days at 4 °C. Before experiments add BSA and 0.5M DDT stock solution (prepare freshly each day) ready to use respiratory solution can be stored for a few hours at room temperature.

The instructions for making ADP, ATP, AMP, and other stock solutions can be found in Table 1. Keep the stock solutions on ice during the experiment.

Sample permeabilization: Tissue permeabilization procedure is advised to carry on as described [14]. For cells, the permeabilization procedure is carried out directly in an oxygraph chamber with saponin for 5 min before starting the measurements. The appropriate saponin concentration should be tested for each sample type i.e., it varies from 25 µg/mL for rat cardiomyocytes [55] to 65 µg/mL for human gastric cancer cell line MKN45 [62]. Permeabilized samples can be stored in Mitomed solution (without respiratory substrates), under gentle shaking on 4 °C for a few hours.

Equipment: Mitochondrial respiration of samples was measured at 25 °C under continuous magnetic stirring with a high-resolution oxygraph (Oxygraph-2 κ Oroboros Instruments, Innsbruck, Austria).

### 3.2. Quantitative Assessment of Intracellular Diffusion Restrictions

Intracellular diffusion restrictions for a certain substrate can be measured indirectly by comparing the apparent *K*m value for the given substrate in permeabilized cells to the corresponding value for isolated enzyme or isolated organelle. Protocol 1 can be used to determine *K*m(ADP) in permeabilized cells using oxygraphy.

**Protocol** **1.** Determination of Km(ADP).

#### Timing ~ 1 h

The *K*m(ADP) which characterizes intracellular diffusion restrictions for ADP, can be determined only in permeabilized tissue samples or cells and not in preparations of isolated mitochondria. The *K*m(ADP) varies significantly between cell types and these differences likely stems from specific structural and functional organization of their energy metabolism. To determine the apparent affinity of mitochondria to exogenous ADP the dependence of respiration rate on exogenous ADP can be measured. From these data, by using Michaelis-Menten equation *K*m for ADP (herein apparent *K*m) and *V*max can be calculated.

1. Add cells/fiber into the oxygraphic chamber.
2. Add respiratory substrates: malate (2 mM) and glutamate/pyruvate (5/10 mM).
3. Register the basal respiration rate (*V*_0_).
4. Start cumulative addition of ADP until the saturation of respiration rate. The ADP concentration range depends on the sample. For preparations with low *K*m(ADP) (e.g., isolated mitochondria and most cell cultures) the concentration range is 0–500 µM ADP. For permeabilized tissue samples the saturating ADP concentration may reach up to 5 mM ADP (usually 2 mM).
5. Calculate the *K*m(ADP) and *V*max values from the [ADP] versus respiration rate (the basal rate of respiration, *K*m _0_, subtracted) relationships on the basis of the Michaelis-Menten equation.

Critical steps: Injectable ADP stock solution should be divided up to eight doses to cover the required concentration range/*K*m curve. Too many additions extends the duration of experiment and thereby constant stirring and reduced oxygen concentration in the chamber could cause mechanical disruption of cell/fiber structure and inactivation of respiration, resulting lower oxygen consumption rates and inaccurate *K*m(ADP) value. Representative traces can be found in [19,26,36,40,63].

Additionally, plotting the data obtained using Protocol 1 to double reciprocal (Lineweaver–Burk) plot gives information about presence of different mitochondrial populations with differently regulated OMM. If the data gives a straight line, the mitochondrial population in the sample is homogeneous.

### 3.3. Quantitative Assessment of Energy-Transfer Pathways

#### 3.3.1. Creatine Kinase Pathway

The stimulatory effects of creatine on mitochondrial respiration allows efficient recycling of ADP inside mitochondria directed by MtCK in IMS and leads to tight coupling of mitochondrial respiration with ATP synthesis (Figure 1). These processes are known and studied in permeabilized cardiac cells over thirty years [64,65]. Further studies have shown that there is a large variability in distribution and the role of CK network between different muscle types and animal species. The increase in the respiration rate in response to creatine addition is 20% in chicken ventricular muscle but for another bird, pigeon, the corresponding number is 60% [66]. The effect of creatine on OXPHOS is well established in rat ventricular cardiomyocytes but not in rat atrial fibers, despite the presence of active MtCK [67]. Interestingly, in human atria MtCK is functionally coupled to OXPHOS, as in ventricular muscle [68].

As was discussed before, one indicator of the level of regulation of respiration kinetics of cells, is the *K*m(ADP). In fast-twitch muscles *m. extensor digitorum longus*, *m. gastrocnemius* white the *K*m(ADP) value is approximately 20 µM, in the same range as isolated mitochondria and it does not change in the presence of creatine [20,21]. In slow-twitch muscles (soleus, heart) with high *K*m(ADP) value it decreases to the 80–100 µM in the presence of creatine [15,21]. In the latter case the phosphotransfer is directed by the CK pathway and therefore the diffusion restrictions for ATP/ADP on OMM level has lower influence on the mitochondrial oxygen consumption rate. Because, in the presence of creatine the functional coupling between MtCK and ANT ensures that ATP synthase uses the ADP circulating in the IMS and therefore the respiration rate increases faster than without creatine. This is reflected in the increase of the mitochondrial apparent affinity to ADP (*K*m(ADP) decreases) [36,42,63,69]. Described dissimilarities in OXPHOS regulation by creatine suggest different roles of CK in these muscles. In fast-twitch glycolytic muscles, the main role of CK is the energy buffering, while in slow-twitch muscles, the CK pathway is responsible for compartmentalized energy transfer. In these cells CK system ensures stable energy supply for myofibrils and ion channel ATPases [4]. In connection with these two tasks, CK network ensures local low ADP level to prevent ATPase inhibition, and proton buffering.

In malignant tumor tissues decrease in the CK activity and creatine content is detected in colon and stomach adenocarcinoma, colon melanoma, as well as skeletal muscle fibrosarcoma cells [29,70]. Interestingly, unlike other CK isoforms, the expression of uMtCK increases in malignant tumor cells. The authors proposed that this phenomenon relates to the uMtCK role as inhibitor of mitochondrial permeability transition pore and therefore apoptosis [70]. Also, in colorectal colon tissue the creatine activation was up to 60% from the exogenous ADP activated maximal respiration, while in corresponding tumor tissue no activation after creatine addition was detected [29].

Here we introduce three protocols developed to characterize the role of CK pathway in energy metabolism using oxygraphic method (Protocols 2–4).

**Protocol** **2.** Determination of Km(ADP) in the presence of creatine.

##### Timing ~ 1 h

1. Intracellular diffusion of adenine nucleotides could be restricted (characterized by high *K*m(ADP) measured in permeabilized tissue/cells) but creatine/PCr transport through the VDAC could bypass the restrictions when CK pathway is functionally coupled to OXPHOS.
2. Add creatine (10 mM) into the respiration media.
3. Add cells/fiber into the oxygraphic chamber.
4. Add respiratory substrates: malate (2 mM) and glutamate/pyruvate (5/10 mM).
5. Register the basal respiration rate *V*_0_.
6. Start cumulative addition of ADP until to respiration rate saturation.
7. Calculate the *K*m(ADP) and *V*max values from the [ADP] versus respiration rate value (the basal rate of respiration, *V*_0_, subtracted) relationships on the basis of the Michaelis-Menten equation. When the calculated *K*m(ADP) value with creatine is significantly lower than the corresponding value without creatine, it confirms an effective functional coupling of OXPHOS to CK pathway.

Critical step: Creatine has low solubility at high concentrations. There is two ways to add ceratine into the oxygraph chamber. The first is to weigh the required amount of substance and add it directly to the chamber. The second is to use creatine stock solution (0.2 M) and keep it at 60 °C. Prepare the injection solution of creatine (0.2 M) just before the experiment. Wash the syringe immediately after every injection to avoid blockage by insoluble residue.

Next protocol (Protocol 3) enables the quantitative measurement of the CK pathway contribution to the energy-transfer flux. With this simple protocol we can see what relative proportion of CK is connected pathway from the entire energy transport in a particular tissue type. For example, in adult mammalian heart muscle cells CK has a strong control over energy transport and OXPHOS while in postnatal heart cells activation of respiration with creatine is not detectable [54]. The following test can only be performed with permeabilized tissue/cell samples because they have intact mitochondria in their natural milieu including ATPases and CK near them which enables functional coupling between ATPases and CK.

**Protocol** **3.** Test for evaluation of the activity of the CK pathway in energy transport and ATP/ADP flux in general.

##### Timing 0.5 h

The test allows assessing functional coupling between the CK pathway and OXPHOS without hampering ATP/ADP diffusion between mitochondria and ATPases.

1. Add cells or permeabilized tissue sample into the oxygraph chamber.
2. Add general respiratory substrates malate (2 mM) + glutamate/pyruvate (5/10 mM) in the oxygraphic chamber and register the basal respiration (*V*_0_).
3. Add MgATP (2 mM) to induce maximal activity of ATPases (V_ATP_). Slight oxygen consumption could be detected in these conditions in resting muscle cells.
4. Add creatine to a final concentration of 20 mM (V_Cr_). If there is a marked rise in respiratory rate after the addition of creatine then CK pathway is activated and concomitant increase in respiration rate reflects functional coupling between mitochondrial CK with OXPHOS as well as general ADP transport activity.

###### Optional:

5. Add ADP (2 mM) to register maximal ADP dependent oxygen consumption rate.

The extent of creatine activation (the creatine index) could be calculated as (V_Cr_ − *V*_0_)/*V*_max(ADP)_

Critical steps**:** Prepare the injection solution of creatine (0.2 M) just before the experiment and keep it at 60 °C because of low solubility of creatine at that concentration. Wash the syringe immediately after every injection to avoid blockage by insoluble residue. Representative traces can be found in [42].

To study specifically the CK pathway and determine the role of MtCK in it the Protocol 4 could be used.

**Protocol** **4.** Creatine test to determine the coupled state of mitochondrial creatine kinase.

###### Timing 0.5–1 h

In this protocol energy flux from the transfer through CK pathway and direct ATP transport could be measured separately. For that purpose, the pyruvate kinase/phosphoenol pyruvate (PK/PEP) system is added to trap extramitochondrial ADP. Therefore, all the ADP produced in ATPase reactions and not engaged in the CK pathway is regenerated by PK/PEP system; and only ADP/ATP circulating inside the mitochondrion activates respiration.

1. Insert cells/fiber into the oxygraphic chamber in addition to the respiratory solution supplemented with substrates: malate (2 mM) and glutamate/pyruvate (5/10 mM) and PEP (5 mM)
2. Add MgATP (2 mM) to activate ATPases. The increase in respiration rate is observable because ADP generated by ATPases is diffused to mitochondria.
3. Add PK (10 U/mL) to activate PK/PEP system which is included to rephosphorylate ADP produced by cytosolic ATPases. While the CK pathway is not activated, energy transport between mitochondrion and ATPases is prevailing and taking place through direct ATP/ADP transfer. Therefore, addition of PK/PEP decreases oxygen consumption rate. In this situation ADP, formed by the ATPases, is regenerated by PK/PEP and backflow of the ADP to mitochondrion is smaller, and oxygen consumption rate, used for rephosphorylation inside mitochondrion, decreases (Figure 1).
4. Start stepwise addition of creatine until saturation is reached (when no additional increase in the respiration is detected). If mitochondrial CK is coupled to OXPHOS, then the respiration in the presence of PK/PEP system is initiated only by ADP generated in mitochondrial intermembrane space by mitochondrial CK.

Critical steps: Prepare the injection solution of creatine (0.2 M) just before the experiment and keep it at 60 °C because of low solubility of creatine at those concentrations. Wash syringe immediately after every injection to avoid blockage by insoluble residue. Representative traces can be found in [19,36,40].

Activation of CK pathway by creatine is very sensitive to the cell/fiber permeabilization quality. Therefore, the appropriate quality tests for outer and inner mitochondrial membrane should be performed in parallel with CK coupling experiments to exclude changes due to poor sample preparation.

The activation rate of CK pathway is decreased in several pathologic conditions in comparison to respective healthy tissues. In human gastric mucosal tissue, an active inflammation weakens coupling between CK and OXPHOS as compared with cells with non-active inflammation [6]. However, there are different tendencies in malignant cells—while decreased levels of creatine and CK is reported, in some cases elevated activities are found [71]. Alterations in the CK pathway activity often appear as a first step before more profound changes of energy metabolism. Also, increase in mitochondrial density is observed in response to the CK/PCr circuit inhibition [72]. In oxidative muscles the need for functional energy transport and buffering at the moments of high energy need is especially important. Therefore, the decrease in CK system has a great impact on muscle performance and a decrease in the function for which this system could be used, to detect outset and progression of pathology.

The activation of respiration as a response to creatine addition is dependent of the complexes functionally connected with the VDAC in the OMM. If the activation of creatine decreases, it is a first sign that complexes, connected with the OMM and regulating VDAC permeability, are partly detached and more molecules of ATP are diffusing to the cytosol. The alterations of CK system are very sensitive and can be detected already before the changes of other kinetic parameters of the OXPHOS.

#### 3.3.2. Adenylate Kinase Pathway

AK catalyzes adenine nucleotide interconversion (2ADP ↔ AMP + ATP) and thereby regulates nucleotide ratios in various cellular compartments, the activity of AMP-sensitive metabolic enzymes, participates in the purine nucleotide synthesis pathway and in regeneration of other nucleoside diphosphates from NTP using AMP as a preferred phosphate substrate [49,73]. Besides, through its unique property of transferring and providing for use both β- and γ-phosphoryl groups of ATP, AK doubles the energetic potential of the ATP molecule. To date, in vertebrates nine AK isoforms (marked as AK1–AK9) have been identified with sub-cellular locations of AK1, AK5, AK7, AK8, AK9 in the cytoplasm, AK2, AK3, AK4 in mitochondria and AK6, AK5, AK9 in the nucleus (reviewed in [73]). Such intracellular placement of different AK isoforms over the entire cell could form an intracellular network for transport of energy-rich phosphoryls between cellular compartments for ensuring efficient feedback between energy consumption and production [7,46,49]. The coupling of OXPHOS with AK system is known to supply energy for a nuclear transport [74]. Proteomics studies have revealed up-regulation of AK2 in human prostate and pancreatic cancer cells [75,76]. It was shown that AK2 can promote cell proliferation under normal circumstances and high expression of AK2 can be associated with poorly differentiated cells with high proliferative index, and that strong differences exist between highly differentiated and tumor cells in the affinity of their mitochondrial respiration for exogenous AMP [49,77,78]. The signaling function of AK realizes through the amplifying a small change in the ATP/ADP ratio into a much higher increase in the AMP/ATP ratio that in turn activates several cellular AMP-sensitive components, including those in the glycolytic and glycogenolytic pathways, and metabolic sensors and effectors such as ATP-sensitive potassium channels and AMP-activated protein kinase, which adjust energy state in the given tissue [1,49,79].

The Protocol 5 enables the determination of the potential of AK system to activate respiration in general. In tissues such as human breast cancer which have low mitochondrial respiration (RCI < 2), only total AK-mediated respiration can be measured by the respirometry method without the addition of PK-PEP system (protocol 5) [29]. Besides, the data obtained by conducting oxygraphic method on human breast cancer, healthy colon, and colorectal cancer (clinical postoperative samples) correlate well with total AK activity in these tissues [29]. This result indicates that the rapid and simple oxygraphic methods can be used to detect changes in AK activity in clinical postoperative tissues.

**Protocol** **5.** Analysis of OXPHOS coupling to AK pathway.

##### Timing 0.5 h

Cells in which AK is functionally coupled to mitochondrial OXPHOS a small decrease in ATP e.g., in case of cellular stress induces a large increase in AMP which stimulates OXPHOS through AK-catalyzed ADP regeneration in mitochondria. This protocol enables determination of the potential of AK to activate respiration in cell cultures, clinical material and in experimental animal preparations. In oxidative muscle cells AMP significantly stimulates respiration at maximal concentration of ADP generated by the system. This reflects the intracellular metabolic compartmentalization and local production of ADP by mitochondrial AK functionally coupled with ANT (Figure 1).

1. Add cells/fiber into the oxygraphic chamber.
2. Add respiratory substrates malate (2 mM) + glutamate/pyruvate (5/10 mM). Register the basal respiration rate.
3. Add MgATP (2 mM or 0.1 mM) to activate ATPases and induce maximal endogenous (intra-systemic) ADP production which should increase the respiration rate.
4. Add AMP (2 mM) to activate the AK reaction and register V_AMP_. Respiration should increase due to activation of cytosolic and mitochondrial AKs. The extent to which respiration is stimulated by AMP indicates the functional coupling of whole AK pathway.
5. Inhibit AK with diadenosine pentaphosphate (AP5A, 0.2 mM, V_AP5A_) in order to measure AK-dependent part of AMP activated respiration. Consequently, in this setup the inhibitory effect of PK on the AMP-mediated O_2_ consumption correlates with intracellular AK1/AK2 ratio.
6. Add carboxyatractyloside (CAT, 1 µM) to inhibit ATP/ADP transport through ANT. In intact mitochondria the respiration is controlled by ANT and if inner mitochondrial membrane is disrupted ANT does not control respiration.
7. To express the strength of the AK functional coupling with OXPHOS calculate AK index (IAK) as IAK = (V_AMP_ − V_AP5A_)/V_AP5A_.

Critical steps: Cells with a low *K*m(ADP) should be measured at low (0.1 mM) ATP concentrations, while for cells with higher *K*m(ADP) vales the use of higher (2 mM) ATP concentrations is recommended [77]. Representative traces can be found in [30,77].

In a more targeted approach, a simple new oxygraphic method was used for quantitative estimation of cellular compartmentalization of AK activity in permeabilized mammalian cells and tissues [77]. The protocol distinguishes between the mitochondrial AK2-dependent respiration activity and activation of respiration induced by the cytosolic AK activity, which is mainly dependent on AK1 activity. The main advantage of this method is its capacity to estimate the relative ratio of AK1 and AK2 activities in one sample without extraction of cellular proteins.

**Protocol** **6.** Determination of AK1 (cytosolic AK) and AK2 (mitochondrial AK) dependent portion of the AMP stimulated respiration.

##### Timing 45 min–1 h

Assessment of AK1/AK2 ratio gives more detailed information about organization of cellular energetic metabolism. AK as the processor of AMP has an influence on regulation of intracellular signaling. Shift in AK1/AK2 may indicate also to the problems in cell differentiation because AK1 is predominating in well differentiated cells.

This is a simple oxygraphic semi-quantitative analysis for the presence of AK1 and AK2 in permeabilized cells. It is based on ATP/AMP-stimulated AK-catalyzed reactions providing ADP to OXPHOS, ADP trapping in the bulk phase of the cytoplasm by the PEP/PK system and measurements of O_2_ consumption rates.

1. Insert cells/fiber into the oxygraphic chamber.
2. Add respiratory substrates malate (2 mM) + glutamate/pyruvate (5/10 mM) to the respiratory solution supplemented with 5 mM PEP. Monitor the basal respiration rate.
3. Add MgATP (2 mM) to induce maximal endogenous (intra-systemic) ADP production.
4. Add AMP (2 mM) to activate the AK reaction coupled with OXPHOs and mediated by AK2 and AK1 and ANT. Register the maximal AMP stimulated respiration (V_AMP_).
5. Injection of 10 IU/mL PK decreases the respiration to the level of AK2 coupled reaction. Because the PEP/PK system is formed and the present V_PK_ demonstrates AK2-specific coupled reaction with ANT inside mitochondria.
6. Add AP5A (0.2 mM) to inhibit AK. Respiration rate should fall significantly.
7. Add CAT (1 μM) to check inner mitochondrial membrane (IMM) intactness. With intact IMM ANT controls the respiration and if control is lost the respiration rate with CAT significantly exceeds the basal respiration rate.
8. The functional coupling with OXPHOS system with AK1 activity could be characterized by the corresponding AK index (I_AK1_). The I_AK1_ is calculated according to the following equation: I_AK1_ = ((V_AMP_ − V_PK)_)/(V_AMP_ − V_AP5A_)) × 100%, where V_AMP_, V_PK_ and V_AP5A_ are the rates of O_2_ consumption that were measured in step 4, 5, 6 respectively. Calculate the index for AK2 functional coupling with OXPHOS s as I_AK2_ = 100% − I_AK1_.

Critical steps: The method is limited by poor mitochondrial respiration of an examined bio-material, i.e., by a respiration control index (RCI) below 2 (see also Protocol 5). Representative traces can be found in [77].

In addition, there is a protocol developed to simultaneously determine coupling of CK and AK to OXPHOS (Protocol 7) [6,68,80].

**Protocol** **7.** Determination of functional coupling of AK and CK to OXPHOS.

##### Timing 1.5 h

Effective activation of respiration by using endogenous ADP sources generated by energy transport pathways is characteristic to cells with high diffusion restrictions for adenine nucleotides in the level of outer mitochondrial membrane. With this protocol we can detect functional coupling between two most common phosphotransfer networks and OXPHOS in one sample. This protocol is especially useful when the amount of test material is limited.

1. Add cells/fiber into the oxygraphic chamber.
2. Add respiratory substrates glutamate/pyruvate (5/10 mM) and malate (2 mM).
3. Add MgATP (50–100 µM) to produce a submaximal amount of endogenous ADP to stimulate mitochondria.
4. Add AMP (2 mM) to activate the coupled reaction of mitochondrial AK (AK2) with ANT. In these conditions the rise in respiration rate (V_AMP_) is caused by coupling of AK to OXPHOS.
5. Add AP5A (0.2 mM, V_AP5A_) to inhibit AK.
6. Add creatine (20 mM) to activate coupled reaction between mitochondrial CK and ANT. In these conditions creatine stimulated respiration (V_Cr_) is activated by local generation of ADP in the vicinity of ANT and associated rise in respiration rate indicates the strength of coupling of MtCK.
7. Add ADP (2 mM) for maximum activation of respiration (V_ADP_).
8. Add cytochrome c (Cyt c, 10 µM) for quality control for intactness of outer mitochondrial membrane.
9. Add CAT (1 µM) to check quality of inner mitochondrial membrane.
10. To assess the strength of the functional coupling independently of mitochondrial content in individual preparations, activation of respiration by AMP can be normalized for the respiratory rate registered after addition of AP5A, thus producing the relative index (I_AK_ = V_AMP_ − V_AP5A_/V_AP5A_). The coupling of CK to OXPHOS is characterized by relative index I_CK_ (I_CK_ = V_Cr_/V_ADP_).

Critical steps: Creatine has low solubility at high concentrations (see also Protocol 1). You can open the oxygraph chamber and add creatine as a pre-weighed substance. Representative traces can be found in [6,80].

#### 3.3.3. Coupling of Hexokinase to Oxidative Phosphorylation

Hexokinases (HKs) catalyze the first and the essentially irreversible step of glycolysis, phosphorylating glucose to glucose 6-phosphate (G6P). Hexokinases 1 (HK1) and HK2 can bind to VDAC through its hydrophobic N-terminus [81,82]. It is believed that HK-VDAC interaction facilitates the access of kinase to newly generated ATP and overcomes the restriction that the OMM exerts on the permeability for the adenine nucleotides (Figure 1) and avoids this interaction product inhibition by G6P. Another consequence of HK-VDAC interaction is that it promotes VDAC closure and blocks the mitochondrial Ca^2+^-dependent opening of the mitochondrial permeability transition pore, in association with protecting the cells from entering apoptosis by preventing binding of pro-apoptotic proteins to VDAC [83].

HK2 is a predominant isoform and it is upregulated in many types of tumors associated with enhanced aerobic glycolysis (the Warburg effect) [60,61,84]. Unlike HK1, the HK2 has retained a catalytic activity of the N-terminal domain and this specific feature enables a doubling of the production of G6P [85]. According to the theory proposed by Pedersen and co-workers the overexpression of the VDAC-bound HK2 is a major player in promoting the growth of aggressive cancers and this enzyme represents good target for cancer therapy [86].

Here we introduce the protocol (Protocol 8) where the coupling between OXPHOS and HK2 can be characterized.

**Protocol** **8.** Investigation of the functional coupling between glycolysis and OXPHOS.

##### Timing 40 min

The coupling of mitochondrion-bound hexokinases (HK) with the OXPHOS in permeabilized cells and tissues can be assayed by high-resolution respirometric test. With this we can measure the ability of HK to stimulate OXPHOS by locally-generated ADP in the vicinity of VDAC channel.

1. Add cells/fiber into the oxygraph chamber.
2. Add respiratory substrates glutamate/pyruvate (5/10 mM) and malate (2 Mm).
3. MgATP (0.1–2 mM) (V_ATP_) is added to achieve maximal stimulation of mitochondria with endogenous ADP e.g., ADP produced by the ATPases.
4. Add glucose (10 mM) to activate the HK reaction (V_Gluc_).
5. Add ADP (2 mM) to achieve maximal ADP-dependent respiration rate (V_ADP_).
6. Add Cyt c (10 µM) for outer mitochondrial membrane quality control.
7. Add CAT (1 µM) for inner mitochondrial membrane quality control.
8. The effect of glucose (glucose index) can be calculated as follows: (V_Gluc_ − V_ATP_)/(V_ADP_).

Critical steps: In tissues or cells with low *K*m(ADP) and/or low capacity to produce endogenous ADP e.g., in cancer tissue, 0.1 M ADP can be added instead of 2 mM ADP, to activate endogenous ADP production. See also Protocol 5. Representative traces can be found in [17,29,80].

## 4. Summary

Deeper understanding of energy-transfer profiles gives important information about the variability of bioenergetic regulation in different tissues in health and disease. Measurements of *K*mADP in permeabilized cells and the high value of it is a good indicator of intracellular complexity in terms of energy transport. Regulated metabolite exchange across the OMM through the VDAC is a crucial modulator of energy metabolism in all cells. In rat cardiomyocytes, where only limited amounts of VDAC channels are permeable to ATP/ADP [87] and these cells possess the high *K*m(ADP) value, is direct transfer by ATP/ADP diffusion predominantly substituted by the CK pathway. Moreover, the loss of complexity (normal structure-function relationships), which is manifested as a decrease in *K*mADP value, could be the first indicator of pathological changes taking part in a tissue or cells, as is shown in case of colorectal carcinogenesis [29,88]. Therefore, if we want to study all the factors influencing energy metabolism, and follow alterations emerging during pathology or aging, intracellular diffusion restrictions for ATP/ADP and energy-transfer pathways should be investigated as well.

It is important to mention that the decrease in the activation of the CK system in the presence of creatine is a very sensitive signal that can indicate to the onset of pathological changes or to first signs of energy metabolism alterations due to aging in oxidative muscle cells [55]. Consequently, these protocols presented here could be used for diagnostics purposes to assess the state of health of the working muscle or other tissues. In recent years, significant progress in cancer treatment has taken place, and especially when the malignant tumor has been discovered at an early stage. Therefore, sensitive protocols, enabling detection of the first alterations in the healthy tissue or benign tumor could give information for successful early diagnosis. Besides, if the pathogenic switch mechanism is detected, it is possible to use this knowledge for development of specialized treatments.

Using permeabilized cells and tissue fibers, several pathways and functional interactions of mitochondrion with different complexes can be studied simultaneously. High-resolution respirometry protocols presented here provide quick and compendious results. These protocols allow characterization of functional mitochondria in their normal intracellular position and assembly, preserving essential interactions with other organelles. As only a small amount of tissue is required for analysis, the protocols can be used in diagnostic settings in clinical studies. It is not with less importance that the results could be acquired with short period of time; the permeabilization procedure and specific analysis can be completed in 2 h.

In conclusion, systemic functional analysis of changes in cellular phosphotransfer networks may help to explain many pathogenic mechanisms in numerous diseases.

## Figures and Tables

**Table 1:** Selected chemicals used in high-resolution respirometry experimental protocols to study creatine kinase and adenylate kinase energy-transfer networks.

Chemical	Stock Concentration (Solvent)	Notes	Storage (°C)
ADP	0.2 M (water)	Adjust pH to 7.1 with KOH	−80, for a short time −20
MgATP	0.2 M (0.1 M HEPES buffer)	Add 0.2 M MgAc 4 H_2_O, adjust pH to 7.1 with NaOH	−80, for a short time −20
AMP	0.2 M (Mitomed, or 0.1 M HEPES)		−80, for a short time −20
Creatine	0.2 M (water)	Keep the solution at +60 °C to avoid precipitation	Fresh
AP5A	0.02 M (water)		−20
CAT	0.2 mM (water)		−20
Cytochrome c	2 mM (water)		−20

## Abbreviations

CK	creatine kinase
AK	adenylate kinase
OXPHOS	oxidative phosphorylation
IMM	inner mitochondrial membrane
OMM	outer mitochondrial membrane
PCr	phosphocreatine
VDAC	voltage-dependent anion channel
IMS	mitochondrial intermembrane space
HK	hexokinase
ANT	adenine nucleotide translocase
MtCK	mitochondrial creatine kinase
uMtCK	ubiquitous MtCK
MI	Mitochondrial Interactosome
PK	pyruvate kinase
PEP	phosphoenol pyruvate
AP5A	diadenosine pentaphosphate
CAT	carboxyatractyloside
